# Diffusion of Cholera in the Remote Districts of Scotland

**Published:** 1855-06

**Authors:** 


### DIFFUSION OF CHOLERA IN THE REMOTE DISTRICTS OF SCOTLAND.

At the meeting of the Edinburgh Medico-Chirurgical Society, on June 6th, Dr. W. T. Gairdner made a verbal communication on the results of some inquiries in which he had been engaged, with reference to the spread of cholera in the remote districts of Scot-land. He found that the epidemic of 1832 had passed with great rapidity to the extreme north, being apparently propagated along the fishing villages of the eastern coast, as far as Caithness-shire. Aberdeen and Inverness had both been involved in the epidemic; and the latter place had been again attacked, two years after, in 1834, when the greater part of the eastern coast was entirely ex-empt. Dr. Gairdner suspected that the disease had, on this occa-sion, been carried along the Caledonian Canal, possibly from Glasgow or the neighbourhood, but he had not been able to make out distinctly the channel of communication. On the west coast, with the exception of a few cases at Stornoway, and possibly some in the island of Skye, he had reason to believe that no cholera had existed north of the Sound of Mull, nor in any of the Hebrides, with the exceptions just mentioned. (*Association Medical Journal*, June 15th.)

